# Capecitabine in Combination with Standard (Neo)Adjuvant Regimens in Early Breast Cancer: Survival Outcome from a Meta-Analysis of Randomized Controlled Trials

**DOI:** 10.1371/journal.pone.0164663

**Published:** 2016-10-14

**Authors:** Ze-Chun Zhang, Qi-Ni Xu, Sui-Ling Lin, Xu-Yuan Li

**Affiliations:** 1 Department of Diagnosis and Treatment Center of Breast Diseases, Shantou Central Hospital, Affiliated Shantou Hospital of Sun Yat-sen University, Shantou, Guangdong, China; 2 Department of Respiratory Medical Oncology, Cancer Hospital of Shantou University Medical College, Shantou, Guangdong, China; 3 Department of Prevention and Health Care, Cancer Hospital of Shantou University Medical College, Shantou, Guangdong, China; 4 Department of Medical Oncology, Shantou Central Hospital, Affiliated Shantou Hospital of Sun Yat-sen University, Shantou, Guangdong, China; University of Michigan, UNITED STATES

## Abstract

Capecitabine has been investigated in early breast cancer in several studies, but it was undefined that whether it could improve survival. To investigate whether the addition of capecitabine affected survival in patients with early breast cancer, a meta-analysis was conducted and overall survival (OS), disease-free survival (DFS), and toxicity were assessed. The PubMed, Embase databases and the Cochrane Central Register of Controlled Trials were searched for studies between January 2006 and April 2016. Hazard ratios (HRs) with their 95% confidence intervals (CIs), or data for calculating HRs with 95% CI were derived. Seven trials with 9097 patients, consisted of 4 adjuvant and 3 neoadjuvant studies, were included in this meta-analysis. Adding capecitabine showed no improvement in DFS (HR = 0.93; 95% CI, 0.85–1.02; P = 0.12), whereas a significant improvement in OS was observed (HR = 0.85; 95% CI, 0.75–0.96; P = 0.008). A sub-analysis of DFS showed that benefit of capecitabine derived from patients with triple negative subtype and with extensive axillary involvement. Safety profiles were consistent with the known side-effects of capecitabine, but more patients discontinued scheduled treatment in the capecitabine group. Combining capecitabine with standard (neo)adjuvant regimens in early breast cancer demonstrated a significantly superior OS, and indicated DFS improvement in some subtypes with high risk of recurrence. Selection of subtypes was a key to identify patients who might gain survival benefit from capecitabine.

## Introduction

Adjuvant polychemotherapy, mostly a regimen containing anthracycline and taxane, has been proved to reduce recurrence and death rate in breast cancer [1]. Neoadjuvant chemotherapy has no downside if clinical assessment suggests that patients with primary breast cancer will require systemic adjuvant therapy. No significant difference was found in overall survival or disease-free survival for adjuvant versus neoadjuvant chemotherapy [2]. However, the 5-year recurrence rate in early breast cancer is still as high as over 20% [3], which suggests probability for improvement. One approach is to incorporate a new drug into standard regimens.

Capecitabine currently is considered one of the most active drugs available for advanced BC, which has a favorable safety profile. A phase III trial has demonstrated that the addition of capecitabine to the docetaxel advanced or metastatic breast cancer significantly prolonged the progression-free survival and overall survival [4].

In adjuvant or neoadjuvant setting, capecitabine rarely improves disease-free survival or overall survival in most studies [5, 6, 7, 8]. However, the statistical assumption in these studies was probably too optimistic (HR, 0.65–0.78) [5, 6, 7, 8], thus might be underpowered to detect small but meaningful improvement in survival. Therefore, we perform a meta-analysis to investigate whether adding capecitabine to standard adjuvant or neoadjuvant chemotherapy would improve the survival outcomes in early breast cancer.

## Methods

### Study Design and Trial Inclusion Criteria

The present study was a systematic review with meta-analysis based on survival data of randomized controlled trials (RCTs) testing the role of capecitabine as part of the (neo)adjuvant therapy in patients with breast cancer. This study was performed in compliance with the checklist provided by the Preferred Items for reporting Systematic Reviews and Meta-Analyses.

The criteria for inclusion were as follows: (1) prospective phase II or III RCTs investigating capecitabine in the (neo)adjuvant setting of breast cancer; (2) RCTs reporting HRs with 95% CI of DFS and OS, or presenting sufficient data for calculating HRs with 95% CIs.

### Search Strategy

Trials were identified by searching the PubMed and Embase databases and the Cochrane library; by examining the reference lists of published trials, review articles and editorials. Abstracts presenting at the annual meetings of the European Society of Medical Oncology (ESMO) and the American Society of Clinical Oncology (ASCO) were also analyzed. The databases were searched for articles published between January 2006 and April 2016.

The following search terms were used to identify potential cancer trials: “breast neoplasm”; “breast carcinoma” or “breast cancer”. The following search terms were used to identify studies investigating therapy with capecitabine: “capecitabine”, “Xeloda”, “neoadjuvant” and “adjuvant”.

### Data Extraction and Quality Assessment

Data extraction and quality assessment were conducted by two reviewers independently. Disagreement between reviewers was resolved by discussion or a third reviewer. Data extracted from the trials included name of the first author, publication year, trial phase, sample size, regimens, DFS, and OS. The quantitative Jadad scale was used to assess study quality [9].

### Statistical Analysis

The p value, HRs and their 95% CIs, if reported, were directly extracted. Otherwise, events in each arm, p values, or published Kaplan-Meier curves were used to estimate HRs and 95% CIs. The summary HRs and their 95% CIs of all included trails were estimated using a general variance-based method. A statistical test with *p* < 0.05 was considered significant. The analysis was two-tailed. Estimates of the treatment effects and toxicity were obtained from the number of events reported in each arm and combined using Mantel-Haenszel methods [10].

Between-study heterogeneity was estimated using the I-squared statistic [11]. Heterogeneity was considered statistically significant when P<0.05 or I^2^>50%. When there was no statistically significant heterogeneity, a pooled effect was calculated with a fixed-effects model; otherwise, a random-effects model was used.

Results are depicted in all figures as conventional meta-analysis forest plots, where HR<1 corresponds to a lower rate of events in the combination arm. Publication bias was assessed by visual inspection of funnel plots and with Egger’s regression asymmetry test.

All statistical analyses were performed with Stata, version 12.0 software (Stata Corporation, College Station, TX, USA).

## Results

### Study Characteristics

**Fig 1:**
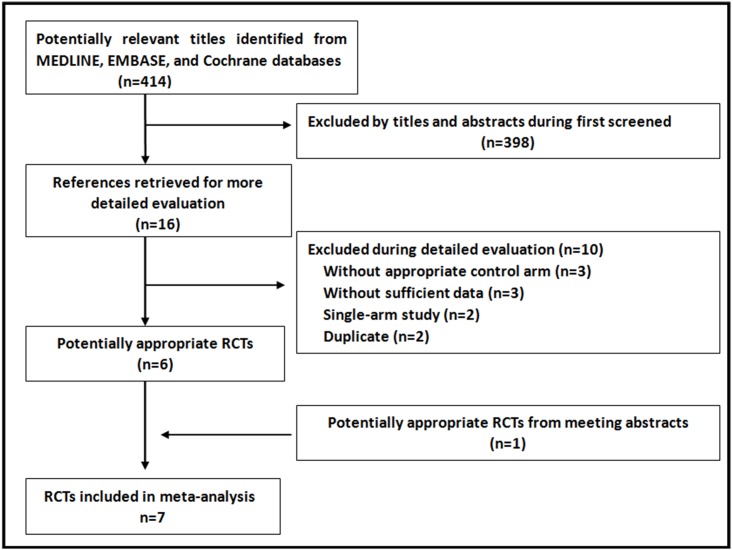
Results of search strategy.

Sixteen potentially eligible trials were identified as full articles for further review. One conference abstract was added [8]. Seven phase III trials met the inclusion criteria of this meta-analysis [5, 6, 7, 8, 12, 13, 14], and 9,097 patients were included in the assessment of OS, DFS, and toxicity. Ten trials were excluded and reasons were listed [15–24], as seen in Fig 1. The funnel plot and Egger's test (*P* = 0.41 and *P* = 0.61 for OS and DFS, respectively) indicated no potential publication bias. The quality was high with all the studies (Jadad score≥3).

Table 1 showed characteristics of the seven randomized trials in this meta-analysis. Table 2 showed patient characteristics of the four adjuvant trials.

**Table 1:** Studies included in the meta-analysis.

Author	Year	Regimens	No.	Dose of capecitabien	Follow-up time	5 year DFS	5 year OS
Joensuu H^[^^5^^]^	2012	TX/CEX	753	1800mg/m2,d1-15	5 years	86.6%	92.6%
		T/CEF	747			84.1%	89.7%
Martin M^[^^6^^]^	2015	ET-X	715	2500mg/m2,d1-14	6.6 years	82.0%	92.0%
		EC-T	669			86.0%	93.0%
O'Shaughnessy J^[^^7^^]^	2015	AC-TX	1307	1650mg/m2,d1-14	5 years	89.3%	94.0%
		AC-T	1304			87.4%	92.0%
Toi M^[^^8^^]^	2015	ATC*	455	2500mg/m2,d1-14	2 years	74.1%	89.2%
		ATC*-X	455			67.7%	83.9%
Bear HD^[^^12^^]^	2015	TX-AC	400	1600mg/m2,d1-14	4.7 years	72.6%	81.5%
		T-AC	394			72.8%	80.9%
von Minckwitz G^[^^13^^]^	2014	EC-TX	479	1800mg/m2,d1-14	5.4 years	52.8%	60.9%
		EC-T-X	471			52.8%	58.2%
		EC-T	471			55.6%	60.9%
Ohno S^[^^14^^]^	2013	FEC-TX	239	1650mg/m2,d1-14	4.5 years	87.9%	95.8%
		FEC-T	238			86.5%	94.9%

X: Capecitabine; T: Docetaxel; C: Cyclophosphamide; E: Epeirubicin; A: Doxorubicin. ATC* = Standard neoadjuvant regimens contains anthracycline, taxanes, or cyclophosphamide.

**Table 2:** Patient characteristics of the four adjuvant trials.

Author	Regimens	Median age	Premenopausal	HR positive	Her2 positive	T1-2	Positive ALN
Joensuu H^[^^5^^]^	3TX-3CEX	52	44.0%	77.0%	19.0%	94.0%	88.0%
	3T-3CEF	53	43.0%	76.0%	19.0%	93.0%	90.0%
Martin M^[^^6^^]^	4ET-4X	51	53.4%	83.1%	9.1%	94.8%	100.0%
	4EC-4T	51	52.2%	85.4%	11.5%	94.6%	100.0%
O'Shaughnessy J^[^^7^^]^	4AC-4TX	50	45.0%	64.0%	12.0%	94.0%	70.0%
	4AC-4T	51	44.0%	64.0%	13.0%	92.0%	69.0%
Toi M^[^^8^^]^	ATC*	48	59.3%	62.9%	0.0%	-	60.2%
	ATC*-8X	48	56.0%	63.9%	0.0%	-	61.3%

X: Capecitabine; T: Docetaxel; C: Cyclophosphamide; E: Epeirubicin; A: Doxorubicin; HR: Hormonal receptor; ALN: Axillary lymph nodes. ATC* = Standard neoadjuvant regimens contains anthracycline, taxanes, or cyclophosphamide.

### Overall Survival and Disease-Free Survival

**Fig 2:**
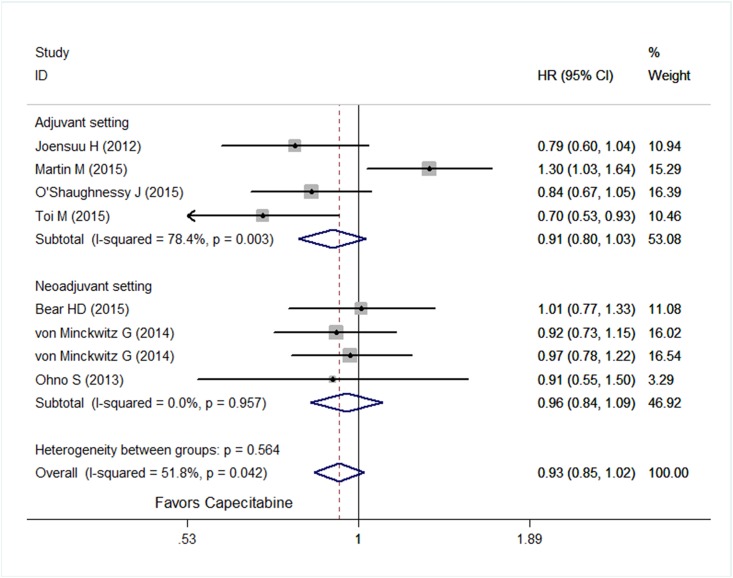
Forest plot of disease-free survival of patients treated with capecitabine versus without capecitabine.

**Fig 3:**
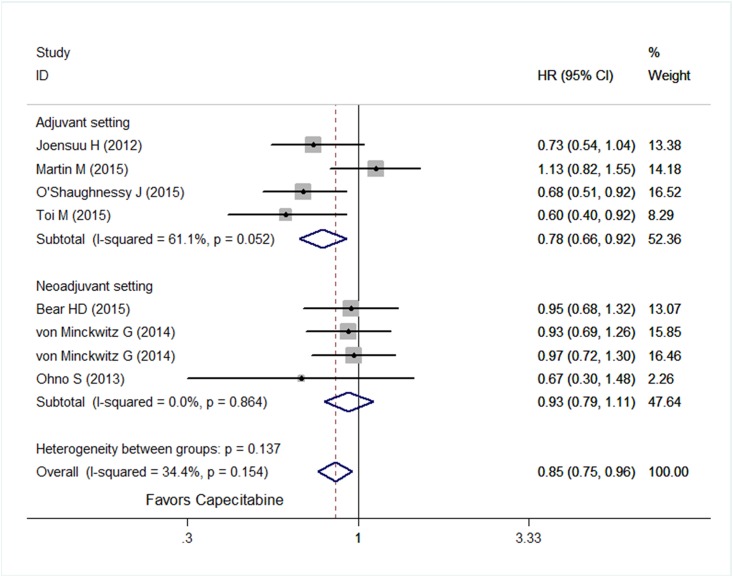
Forest plot of overall survival of patients treated with capecitabine versus without capecitabine.

**Fig 4:**
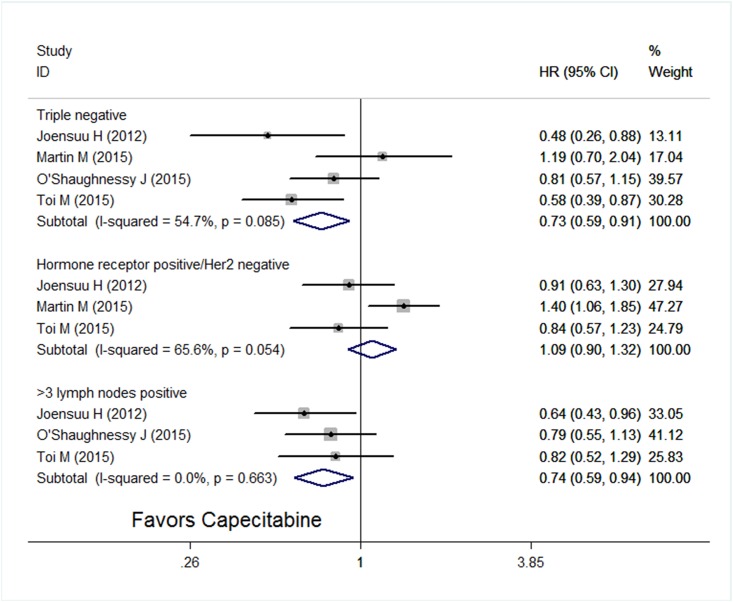
Forest plot of disease-free survival of subgroup analysis in the adjuvant setting.

Considering heterogeneities among included trials and patients, a random-effect model was used. Compared with similar regimens without capecitabine, integration of capecitabine showed no improvement in DFS (HR = 0.93; 95% CI, 0.85–1.02; *P* = 0.12) (Fig 2), whereas a significant improvement in OS was seen when incorporating capecitabine (HR = 0.85; 95% CI, 0.75–0.96; *P* = 0.008) (Fig 3). A sub-analysis of DFS was performed, as shown in Fig 4, adding capecitabine resulted in improvement of DFS in patients with triple negative subtype (HR = 0.73; 95% CI, 0.59–0.91; *P* = 0.005) and with more than three positive lymph nodes (HR = 0.74; 95% CI, 0.59–0.94; *P* = 0.012), whereas no benefit was observed in patients with hormone receptor positive and Her2 negative (HR = 1.09; 95% CI, 0.90–1.32; *P* = 0.36).

### Toxicity

Overall, safety profiles were consistent with the known side-effects of capecitabine. No capecitabine-related death was reported among the included trials. However, there were approximately 24%, 13%, and 22% of patients in the Finxx [5], GEICAM/2003-10 [6], and CREAT-X [8] trials, respectively, discontinued scheduled treatment during the capecitabine-containing therapy.

## Discussion

The role of capecitabine in patients with metastatic breast cancer had been established [25], and studies were further conducted to test its efficacy in the (neo)adjuvant setting. When capecitabine was added into the standard neoadjuvant regimens, there were inconsistent results in terms of pathological response rate [12, 18]; however, a meta-analysis failed to show neoadjuvant treatment with capecitabine had improved pathological response rate [26]. Moreover, results in survival were consistently disappointing although neoadjuvant trials were not designed to detect a survival benefit. In addition, DFS and OS improvement were seen in a few adjuvant trials [7, 8]. A previous meta-analysis investigated the role of capecitabine in the adjuvant setting and concluded that a taxane-anthracycline-capecitabine regimen was effective and tolerated in high-risk early breast cancer [27]. In the context of more and more trials involving treatment with capecitabine in early breast cancer, a meta-analysis was warranted to assess whether adding capecitabine could result in improvement in DFS or OS. The present meta-analysis showed that adding capecitabine into (neo)adjuvant therapy did not improve DFS but ameliorated OS. The toxicity profile of capecitabine remained favorable among trials.

There were some factors that might have contributed to the negative results of DFS. First, the optimal dose of capecitabine as (neo)adjuvant therapy was not yet defined, which ranged from 1600mg/m^2^ to 2500 mg/m^2^ on day 1–14 among trials. Second, reduction of docetaxel was usually required in light of increased toxicity when capecitabine was combined, and this might compromise the effect of the study arm. Third, dose reduction of capecitabine and discontinuation of scheduled treatment, both due to toxicity, could as well affect the outcome.

Conversely, a significant improvement in OS was observed with the capecitabine-containing regimen but the result should be cautiously interpreted. Two included studies reported OS prolongation [7, 8]. In a trial carried out by the US Oncology Group [7], patients assigned to capecitabine-containing also had better overall survival in absence of a DFS benefit. The authors postulated the significant OS results might be, in part, explained by the lower rate of systemic recurrence in the capecitabine-containing treatment. The CREAT-X study used capecitabine as consolidated adjuvant chemotherapy in breast patients who had residual invasive disease after neoadjuvant therapy and DFS and OS were significant improved [8]. Results from the CREAT-X study suggested that efficacy benefit of capecitabine may be limited to early breast cancer with high risk of recurrence.

Some subtypes of early breast cancer such as triple-negative cancer [28] or cancers with extensive axillary involvement, which were associated with a high risk of cancer recurrence, could possibly benefit from capecitabine. In the subgroup analysis for DFS, patients with triple negative subtype and those with more than three positive lymph nodes showed a significant improvement, whereas no benefit was observed in patients with hormone receptor positive and Her2 negative who were deemed to have a low risk of recurrence. In addition, Ki-67 was suggested as a predictive factor to identify responders [8, 14]. Patients with low Ki-67 expression were less likely to benefit from capecitabine. Together these results would provide information on the selection of patients in further (neo)adjuvant trials evaluating capecitabine in the treatment of early breast cancer.

The present meta-analysis has several limitations. Firstly, the number of studies in this study was low and analysis was not based on individual patient data, and data from one trial, which demonstrated positive result in DFS and DS, were reported only in abstract. Secondly, treatment schedules including drug dosage differed among the trials. Thirdly, breast cancer was regarded as a family of diseases, hence patients included in this analysis were considered to have different biology and different prognosis. These differences could cause great heterogeneity. Finally, limited sub-group analysis was unable to provide more information.

In summary, the incorporation of capecitabine into a standard (neo)adjuvant regimen in early breast cancer showed no DFS improvement in all patients except for some subtypes with high risk of recurrence, and demonstrated a significantly superior OS. It is important to select patients with early breast cancer who were mostly likely to have improved outcomes from the use of capecitabine in (neo)adjuvant therapy.

## Supporting Information

S1 Checklist PRISMA checklist. (DOC)

S1 Fig Funnel Plot of Standard Error by Log Hazard Ratio. Endpoint Was Overall Survival.
(TIF)
