# Supplementary figures and images for: Capecitabine in Combination with Standard (Neo)Adjuvant Regimens in Early Breast Cancer: Survival Outcome from a Meta-Analysis of Randomized Controlled Trials
Source: PLoS One. 2016 Oct 14;11(10):e0164663. doi: 10.1371/journal.pone.0164663 (PMC5065157; doi:10.1371/journal.pone.0164663)

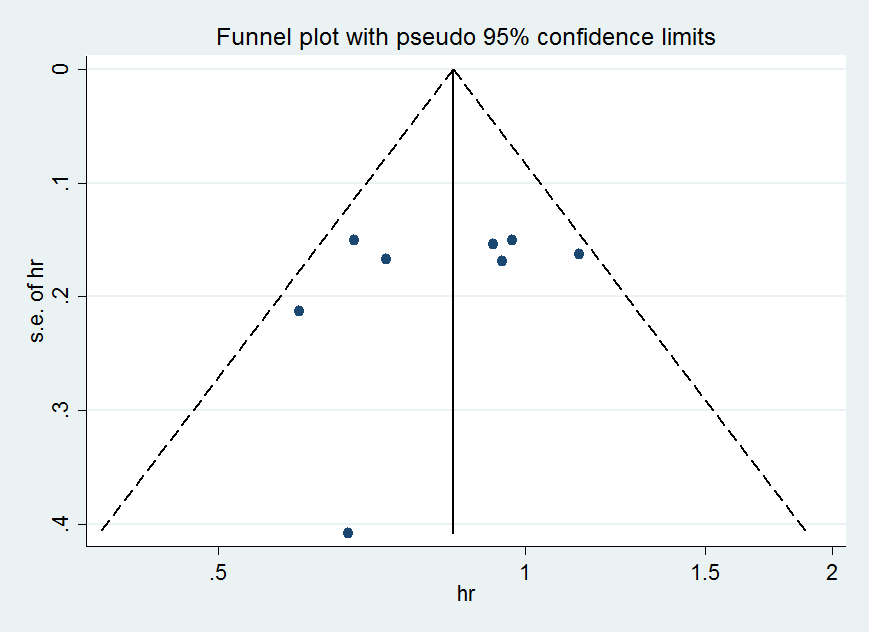

Supplement: S1 Fig — Endpoint Was Overall Survival. (TIF) [file pone.0164663.s002.tif]
